# The complete chloroplast genome sequence of *Paphiopedilum henryanum* (Orchidaceae)

**DOI:** 10.1080/23802359.2022.2088310

**Published:** 2022-06-24

**Authors:** Hang Ye, Hengzhao Liu, Guojia Hu, Peng Zhao

**Affiliations:** Key Laboratory of Resource Biology and Biotechnology in Western China, Ministry of Education, College of Life Sciences, Northwest University, Xi’an, China

**Keywords:** *Paphiopedilum henryanum*, complete chloroplast genome, genetic study

## Abstract

*Paphiopedilum henryanum* Braem 1987 is a critically Endangered terrestrial orchid mainly occurred around the southern China and northern Vietnamese borders. Recently, the population size of this species has been sharply declined due to many threats such as climate change, habitat loss, and narrow distribution. In this study, the complete chloroplast genome sequence of *P. henryanum* was determined from Illumina pair-end sequencing data. The chloroplast genome was 155,886 bp in length, including a large single copy region (LSC) of 87,573 bp, a small single-copy region (SSC) of 2,831 bp, and a pair of inverted repeated regions (IRa and IRb) of 32,741 bp each. The chloroplast genome contained 121 genes corresponding to 76 protein-coding genes, 8 ribosomal RNA (rRNA) genes and 37 transfer RNA (tRNA) genes, respectively. The results of phylogenetic analysis indicated that *P. henryanum* was closely related to *P. violascens* and *P. venustum* in the genus *Paphiopedilum* based on sixteen whole chloroplast genome sequences. The results would provide a valuable resource for future genetic studies of *Paphiopedilum*.

The genus *Paphiopedilum* is a predominant group of orchids which including about 66 species mainly distributed over Asiatic tropics to pacific regions. As an important perennial herbal germplasm resource of Orchidaceae, *Paphiopedilum henryanum* Braem 1987 possesses significant ornamental and medicinal value, grows primarily on the crevices of shaded cliffs or rocky and well-drained places in evergreen broad-leaved forests with an altitude of 700–1400 m spanning southern China (southwest Guangxi, southeast Yunnan) and northern Viet Nam (Xu et al. 2018). However, the increased developments as an economic resource have accelerated the degradation of *P. henryanum* populations so that the population size has declined by 80% over the last decade and up to 90% over the last three generations (25 years). Nowadays this species has been listed as a first-class protected plant in China as well as listed in the IUCN Red List of Critically Endangered (CR) Species Xu et al. 2018).

To provide theoretical basis for formulating effective and reasonable protection strategies, more molecular and genetic resources are urgently needed for further studies of *P. henryanum*. In recent years, researches upon *P. henryanum* mostly focuses on ecological adaptation (Wang et al. 2011) and cultivation (Yang et al. 2019). Furthermore, *Paphiopedilum* was first described by Pfitzer in 1886 followed by infrageneric classifications of the genus proposed by various researchers. There was no unique morphological character to distinguish the slipper orchid genera from each other (Cox et al. 1997). Although molecular phylogenetic analyses over the past two decades have brought much of the deeper-level relationships of *Paphiopedilum* into focus, these studies have relied primarily on nrITS and a handful of plastid genes such as trnL-F and ycf1, and many deep-level relationships remain unclear (Cox et al. 1997; Chochai et al. 2012; Kim et al. 2015). Thus, complete chloroplast accompanied by visible ascendancy has become a meritorious tool for phylogenetic and evolutionary research of *Paphiopedilum* (Dong et al. 2014; Izan et al. 2017). The present study assembled and characterized the complete chloroplast genome of *P. henryanum* based on Illumina pair-end sequencing data. And the results would not only supply fundamental understanding for the development and conservation of the plant germplasm, but also be a useful resource on future genetic researches as well as determining phylogenetic relationships in the genus *Paphiopedilum*.

We guaranteed that we complied with the IUCN, CBD and CITES research policies involving a Threatened/Endangered species. Our study was conducted in accordance with the laws of the People’s Republic of China, and field collection was approved by the Chinese Government. All researchers received permission letters from the College of Life Science, Northwest University, to collect the samples. The cultivar plant sample was collected from the arboretum of Lichuan County Bureau of industry and information technology in Pu'er City, Yunnan Province (100°42′E, 23°29′N) in China and the voucher specimen was stored at the herbarium of Northwest University (108°55′E, 34°15′N, College of Life Sciences, accession number: SK2021201, Hang Ye, SXSDyehang@hotmail.com). The total genomic DNA was extracted from fresh leaves using a modified CTAB method (Doyle and Doyle 1987). The libraries of insert sizes of ∼350 bp were constructed from randomly fragmented genomic DNA, and 150 bp paired-end reads were subsequently produced on the Illumina Hiseq platform (Illumina, San Diego, CA, USA). The GetOrganelle (Jin et al. 2020) software was employed to assemble the filtered reads by using the *P. purpuratum* chloroplast genome sequence as a reference (GenBank accession number: MN535015) (Chen et al. 2019), after which the assembled chloroplast genome was annotated using the online tools GeSeq (Tillich et al. 2017). The accurate new annotated complete chloroplast genome was submitted to GenBank with accession number MN315108.

The complete chloroplast genome of *P. henryanum* was 155,886 bp in length with 35.90% GC contents, containing a pair of inverted repeats (IRs) region of 32,741 bp detached by a large single-copy (LSC) region of 87,573 bp and a small single-copy (SSC) region of 2,831 bp, to exhibit a typical quadripartite structure. There was a total of 121 genes, including 76 protein-coding genes, 8 ribosomal RNA (rRNA) genes and 37 transfer RNA (tRNA) genes. Compared with another accession of *P. henryanum* collected form Guilin (OK514750) in NCBI, the length of the latter was much longer (157,090 bp, consisted of 86,812, 34,220 and 1,838 bp for LSC, IRs and SSC, respectively) with 35.87% GC contents. There were 265 polymorphic sites and 12,968 gaps detected after sequence alignment. The gene contents were also significantly different between the two haplotypes. Compared with our results, there were only 72 protein coding genes in OK514750, in which four protein coding genes including petD, psbJ, and two copies of ycf2 were loss. Moreover, the tRNA gene trnK-UUU were only occurred in MN315108 while the trnL-UAG were unique to OK514750.

The phylogenetic analysis based on complete chloroplast genome sequences was carried out between the two records of *P. henryanum* MN315108 and *P. henryanum* OK514750 as well as 13 other Orchidaceae species (Figure 1) obtained from NCBI. Two Liliaceae species were selected as outgroups. The sequence alignment was implemented using MAFFT software with the default paraments (Katoh and Standley 2013) and the maximum-likelihood (ML) phylogenetic tree was constructed using IQ-TREE (Minh et al. 2020) software with 1,000 bootstrap replicates, in which the nucleotide substation model TVM + F+R3 was adopted as the best-fit model according to the Bayesian Information Criterion (BIC) scores. The result showed that the two *P. henryanum* individuals were clustered into the same clade in the genus *Paphiopedilum* and were most closely related to *P. violascens* and *P. venustum* with 100 bootstrap support value (Figure 1). Furthermore, the three *Cymbidium* species did not gather into the same clade, reflecting relatively high variability. Overall, the complete chloroplast genome resource provided the basis for future research on the evolution and molecular biology of the genus *Paphiopedilum* and Orchidaceae species.

**Figure 1.**
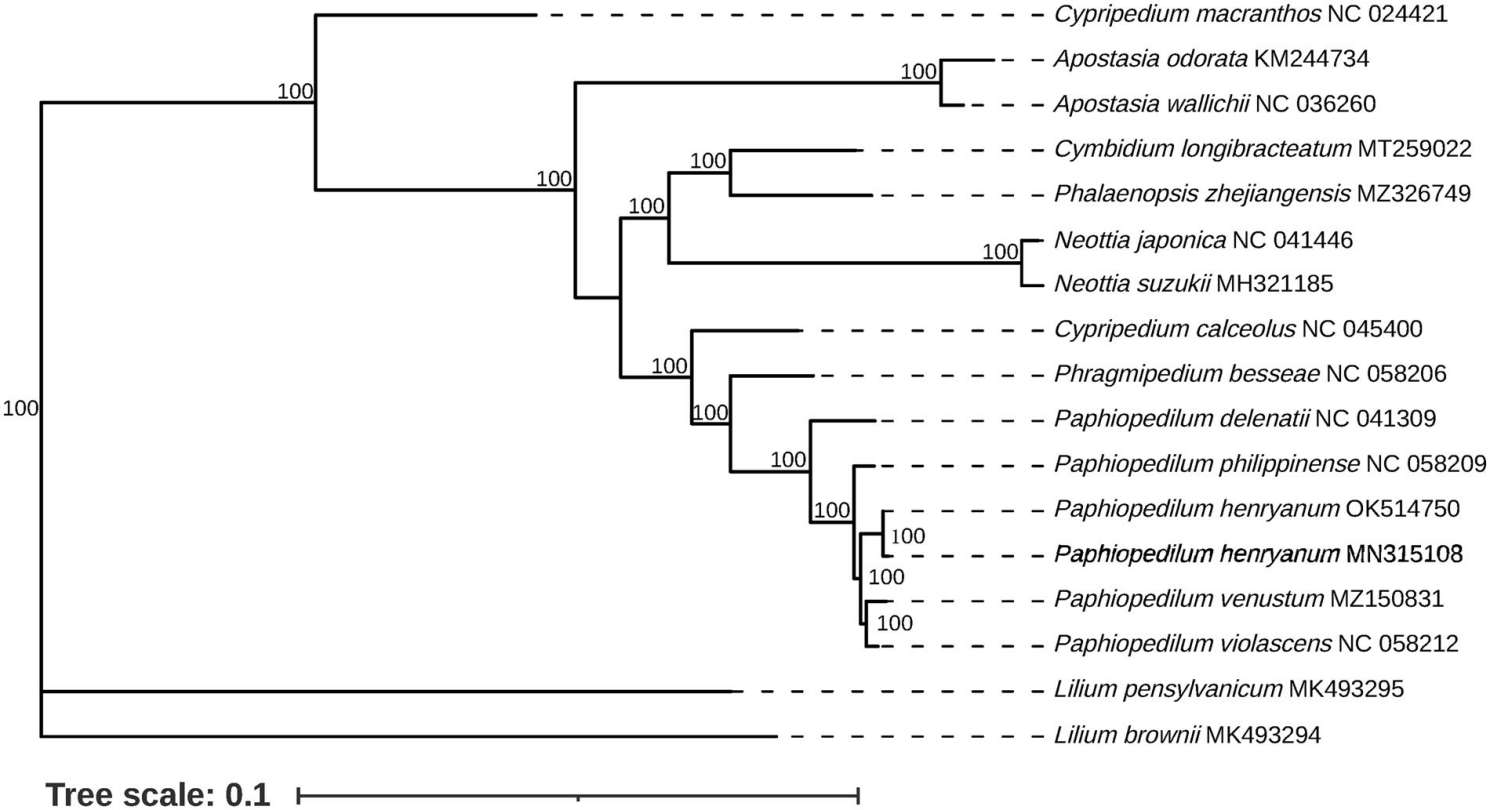
Maximum-likelihood phylogenetic tree for *P. henryanum* MN315108 based on 17 complete chloroplast genomes. The number on each node indicated the bootstrap support value.

## Ethical approval

We guaranteed that we complied with the IUCN, CBD and CITES research policies involving a Threatened/Endangered species. Our study was conducted in accordance with the laws of the People’s Republic of China, and field collection was approved by the Chinese Government. All researchers received permission letters from the College of Life Science, Northwest University, to collect the samples.

## Author contributions

Peng Zhao designed and managed the project; Hang Ye, Hengzhao Liu and Guojia Hu collected the materials and performed data analysis. Hang Ye, and Hengzhao Liu wrote the first draft. Peng Zhao revised the manuscript. All authors read and approved the final manuscript and agreed to be accountable for all aspects of the work.

## Data Availability

The data that support the findings of this study are openly available in NCBI Genbank with the accession number MN315108 (https://www.ncbi.nlm.nih.gov/nuccore/MN315108). The associated BioProject, SRA, and Bio-Sample numbers are PRJNA791767, SRR17318927, and SAMN24344703 respectively.
